# Rising Mortality Related to Diabetes Mellitus and Hypertension: Trends and Disparities in the United States (1999−2023)

**DOI:** 10.1002/clc.70132

**Published:** 2025-04-16

**Authors:** Shahnawaz Hashmi, Iqra Safdar, Muhammad Hazqeel Kazmi, Eeshal Zulfiqar, Maryam Shahzad, Sonia Hurjkaliani, Mennatalla Ayyad, Nimra Zuberi, Toqeer Ahmed, Gauri Balan Sujay, Hamid Talal, Syeda Hazqah Kazmi, Muhammad Farooq Khan, Gabriel Imbianozor, Mushood Ahmed, Raheel Ahmed

**Affiliations:** ^1^ James Cook University Hospital Middlesbrough UK; ^2^ South Tyneside and Sunderland NHS Trust Sunderland UK; ^3^ Dow University of Health Sciences Karachi Pakistan; ^4^ Doncaster Royal Infirmary Doncaster South Yorkshire UK; ^5^ Quaid e Azam Medical College Bahawalpur Pakistan; ^6^ Sunderland Royal Hospital Sunderland UK; ^7^ Rawalpindi Medical University Rawalpindi Pakistan; ^8^ Royal Brompton Hospital London UK; ^9^ National Heart & Lung Institute, Imperial College London UK

**Keywords:** diabetes mellitus, hypertension, mortality, United States

## Abstract

**Background:**

Individuals with diabetes mellitus (DM) are at an increased risk of vascular stiffness and atherosclerosis, which can predispose them to hypertension (HTN). Our study aims to analyze long‐term mortality trends related to DM and HTN in the United States (US) and to identify vulnerable populations.

**Methods:**

The CDC WONDER database was used to extract mortality data among adults (≥ 25 years of age) in the US who had concomitant DM and HTN. Age‐adjusted mortality rates (AAMRs) were estimated and mortality trends were assessed using annual percentage change (APCs) with JoinPoint.

**Results:**

A total of 2 769 118 deaths were attributed to DM and HTN in the US from 1999 to 2023. The AAMRs increased from 14.9 in 1999 to 66.8 in 2023 reflecting a 4.5‐fold increase in mortality. A peak in mortality was observed during the COVID‐19 pandemic with AAMR reaching 77.9 in with an APC of 15.7. Men had consistently higher AAMR compared to women (84.5 vs. 52.6 in 2023). Among racial/ethnic groups, non‐Hispanic (NH) Black or African American individuals had the highest average AAMR, followed by Hispanic or Latino individuals, NH Other populations, and lastly the NH White individuals. The south had the highest AAMR among census regions and rural areas had higher mortality rates compared to urban areas (85.5 vs. 71.7).

**Conclusion:**

Our study shows a 4.5‐fold increase in DM and HTN‐related mortality in the United States from 1999 to 2023. Demographic and geographical disparities were evident with men, NH Blacks or African Americans, and rural areas at the highest risk reflecting the need for improved healthcare.

## Graphical abstract

Diabetes and hypertension‐related mortality increased 4.5‐fold in the United States from 1999 to 2023, peaking during the COVID‐19 pandemic. Men, non‐Hispanic Black individuals, and rural populations experienced the highest mortality rates, highlighting significant disparities. Targeted public health interventions are needed to address the rising burden and healthcare inequities in vulnerable populations.

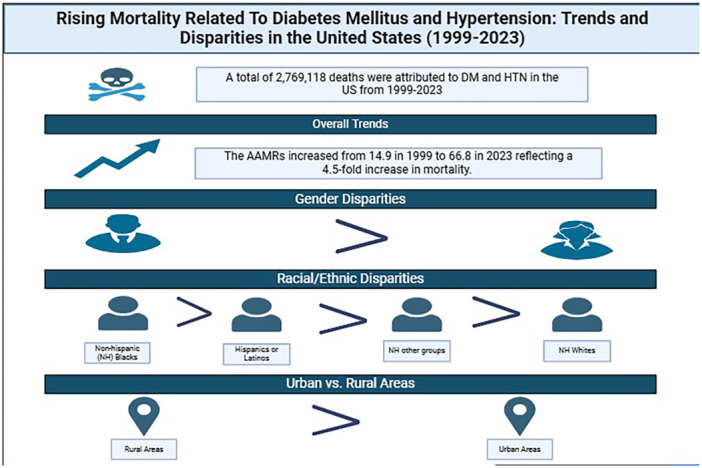

## 1 Introduction

Diabetes mellitus (DM) and hypertension (HTN) rank among the most common chronic diseases worldwide [1]. Approximately 15% of the population in the United States (37 million individuals) are affected by diabetes, while nearly half of US adults (49.6%, or 115 million persons) are afflicted with HTN [2, 3, 4, 5]. This substantial disease burden has significant public health implications and is associated with poor prognosis. The primary causes of death in the United States include cardiovascular disease, kidney failure, and stroke which are directly linked to DM and HTN [3, 4]. Previous studies have shown that almost 50%−80% of individuals with diabetes have concomitant HTN [6, 7]. Chronic hyperglycemia in DM induces vascular endothelial dysfunction, which accelerates the development of HTN. Other reasons include maladaptive changes in the autonomic nervous system, enhanced renin‐angiotensin‐aldosterone system (RAAS) activation, and immune function alterations [8, 9].

While previous studies have explored mortality disparities in patients with DM and HTN separately, none have specifically examined mortality in individuals with co‐existing DM and HTN, a population at higher risk for worse clinical outcomes [10, 11, 12, 13, 14, 15, 16]. Furthermore, no prior studies have assessed the impact of COVID‐19 on this vulnerable group. Comprehending the inequities related to DM and HTN‐related mortality is critical in identifying high‐risk cohorts and formulating focused therapies and targeted lifestyle changes. Considering this literature gap, our study aimed to assess DM and HTN‐related mortality in the United States from 1999 to 2023, and identify populations at heightened risk.

## 2 Methods

### 2.1 Study Setting and Population

Deaths occurring within the United States related to DM and HTN were extracted from the CDC WONDER (Centers for Disease Control and Prevention Wide‐Ranging ONline Data for Epidemiologic Research) database [1]. CDC‐WONDER is an exhaustive repository of death certificate data from the 50 states of the USA as well as the District of Columbia. The Multiple Cause‐of‐Death Public Use record death certificates were studied to identify records in which DM and HTN were reported as multiple causes of death on nationwide death certificates. Patients were identified using the International Classification of Diseases 10th Revision Clinical Modification (ICD‐10‐CM) codes E10‐E14 for DM and I10‐I15 HTN in individuals ≥ 25 years of age. These codes are validated and have been used in prior administrative database studies [17, 18]. Institutional review board approval was not required for this study, as we used a publicly available, de‐identified data set provided by the government. The study adheres to the reporting standards outlined in the Strengthening the Reporting of Observational Studies in Epidemiology (STROBE) guidelines [19].

### 2.2 Data Abstraction

Data on DM and HTN‐related deaths and population sizes were extracted. Demographics (sex, race/ethnicity, and age), and regional information (urban‐rural and state) were extracted from 1999 to 2023. Race/ethnicities were delineated as non‐Hispanic (NH) White, NH Black or African American, NH others (American Indian or Alaska Native, Native Hawaiian or Pacific Islander, Asian, More than one race), and Hispanics or Latinos. These race/ethnicity categories have previously been used within analyses from the CDC WONDER database and rely on reported data on death certificates [20, 21]. Trends in mortality from DM and HTN were evaluated based on state‐specific variations, US census regions (Northeast, Midwest, South, West), and county‐level urbanization classifications. Counties were categorized as rural (micropolitan, noncore regions) or urban (large central metro, large fringe metro, medium metro, small metro) based on the 2013 National Center for Health Statistics Urban‐Rural Classification Scheme [22]. Similar stratification has been used in prior research [21, 23, 24, 25].

### 2.3 Statistical Analysis

Crude and age‐adjusted mortality rates (AAMRs) per 100 000 population were determined. Crude mortality rates (CMRs) were determined by dividing the number of DM and HTN‐related deaths by the corresponding US population of that year. AAMRs were calculated by standardizing the DM and HTN‐related deaths to the 2000 US population as previously described [26]. The Joinpoint Regression Program (Joinpoint V 5.1.0.0, National Cancer Institute) was used to determine trends in AAMRs and CMRs using annual percent change (APC) [27]. This method identifies significant changes in AAMRs and CMRs over time by fitting log‐linear regression models where temporal variation occurred. APCs with 95% confidence intervals (CI) for the AAMRs and CMRs were calculated at the identified line segments linking join points using the Monte Carlo permutation test. APCs were considered increasing or decreasing if the slope describing the change in mortality was significantly different from zero using 2‐tailed *t* testing. Statistical significance was set at *p* < 0.05.

## 3 Results

### 3.1 Overall Trends

**Figure 1:**
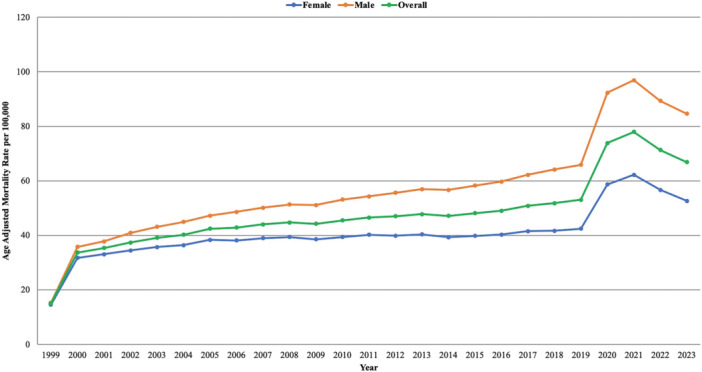
Overall and sex‐stratified age‐adjusted mortality rates (AAMRs) per 100,000 individuals in the United States, 1999 to 2023.

From 1999 to 2023, a total of 2 769 118 DM and HTN‐related deaths were reported among adults in the United States. The AAMR rose significantly, from 14.94 in 1999 to 35.35 in 2001, with an APC of 43.99* (95% CI: 19.87 to 61.06; *p* < 0.000001). This was followed by a further increase in AAMR to 51.79 in 2018 with an APC of 1.60* (95% CI: 0.85 to 2.13; *p* = 0.0032). The AAMR again rose sharply from 51.79 in 2018 to 77.93 in 2021 reflecting an APC of 15.72* (95% CI: 10.33 to 19.33; *p* = 0.0004), followed by a significant decline to 66.88 in 2023 with an APC of −8.44* (95% CI: −13.54 to −2.19; *p* = 0.016). (Supporting Information S1: Table 1 and Supporting Information S1: Table 2, Figure 1).

### 3.2 DM and HTN‐Related Mortality Trends Stratified by Sex

From 1999 to 2023, males averaged a considerably higher AAMR than females. For males, the AAMR increased significantly from 15.27 in 1999 to 37.82 in 2001, with an APC of 45.80* (95% CI: 19.05 to 65.16; *p* < 0.000001). The AAMR further rose to 64.13 in 2018 with an APC of 2.38* (95% CI: 1.58 to 2.94; *p* = 0.0012). From 2018 to 2021, the AAMR further increased to 96.86 reflecting an APC of 16.06* (95% CI: 10.82 to 19.64; *p* = 0.0004). The AAMR then decreased from 96.86 in 2021 to 84.58 in 2023 with an APC of −7.93* (95% CI: −12.80 to −1.89; *p* = 0.016).

The AAMR for females also increased significantly from 1999 to 2001, from 14.54 to 33.05, with an APC of 40.18* (95% CI: 17.30 to 56.18; *p* < 0.000001). This was followed by a stable trend from 2001 to 2018. The AAMR again rose from 41.66 in 2018 to 62.23 in 2021 with an APC of 15.19* (95% CI: 9.35 to 19.04; *p* = 0.002). The AAMR then declined sharply to 52.61 in 2023, reflecting an APC of −8.96* (95% CI: −14.42 to −2.002; *p* = 0.019). (Supporting Information S1: Table 2 and Supporting Information S1: Table 3, Figure 1).

### 3.3 DM and HTN‐Related Mortality Trends Stratified by Race/Ethnicity

Throughout the study period, NH Black or African American individuals had the highest average AAMR, followed by Hispanic or Latino individuals, NH Other populations, and lastly the NH White individuals.

For NH Black or African American group, the AAMR increased significantly from 42.64 in 1999 to 88.76 in 2001 with an APC of 37.95* (95% CI: 13.22 to 55.25; *p* < 0.000001), then remained stable till 2018. From 2018 to 2021, the AAMR rose significantly from 95.45 to 141.73, with an APC of 16.66* (95% CI: 9.85 to 21.35; *p* = 0.002). This was followed by a sharp decline in AAMR to 117.24 in 2023, reflecting an APC of −11.97* (95% CI: −18.06 to −4.30; *p* = 0.0096).

For Hispanic or Latino, the AAMR also increased significantly from 19.91 in 1999 to 49.62 in 2001, reflecting an APC of 43.97* (95% CI: 2.83 to 79.12; *p* = 0.0068), followed by a stable trend through 2018. The AAMR again rose sharply from 61.44 in 2018 to 97.96 in 2021, with an APC of 19.56* (95% CI: 11.17 to 26.19; *p* < 0.000001). This was followed by a significant decline in AAMR to 76.38 in 2023, with an APC of −16.80* (95% CI: −24.06 to −6.80; *p* = 0.0064).

The AAMR for NH Others population initially increased significantly from 16.00 in 1999 to 35.91 in 2001 with an APC of 41.26* (95% CI: 10.68 to 65.24; *p* < 0.000001). Following this peak, the AAMR remained stable from 2001 to 2018. This was followed by an increase from 2018 to 2021, as the AAMR rose from 48.1 to 70.86 with an APC of 15.88* (95% CI: 10.31 to 19.65; *p* < 0.000001). The AAMR then declined significantly from 2021 to 2023, as the AAMR decreased to 57.74 with an APC of −11.92* (95% CI: −17.07 to −5.85; *p* = 0.002).

**Figure 2:**
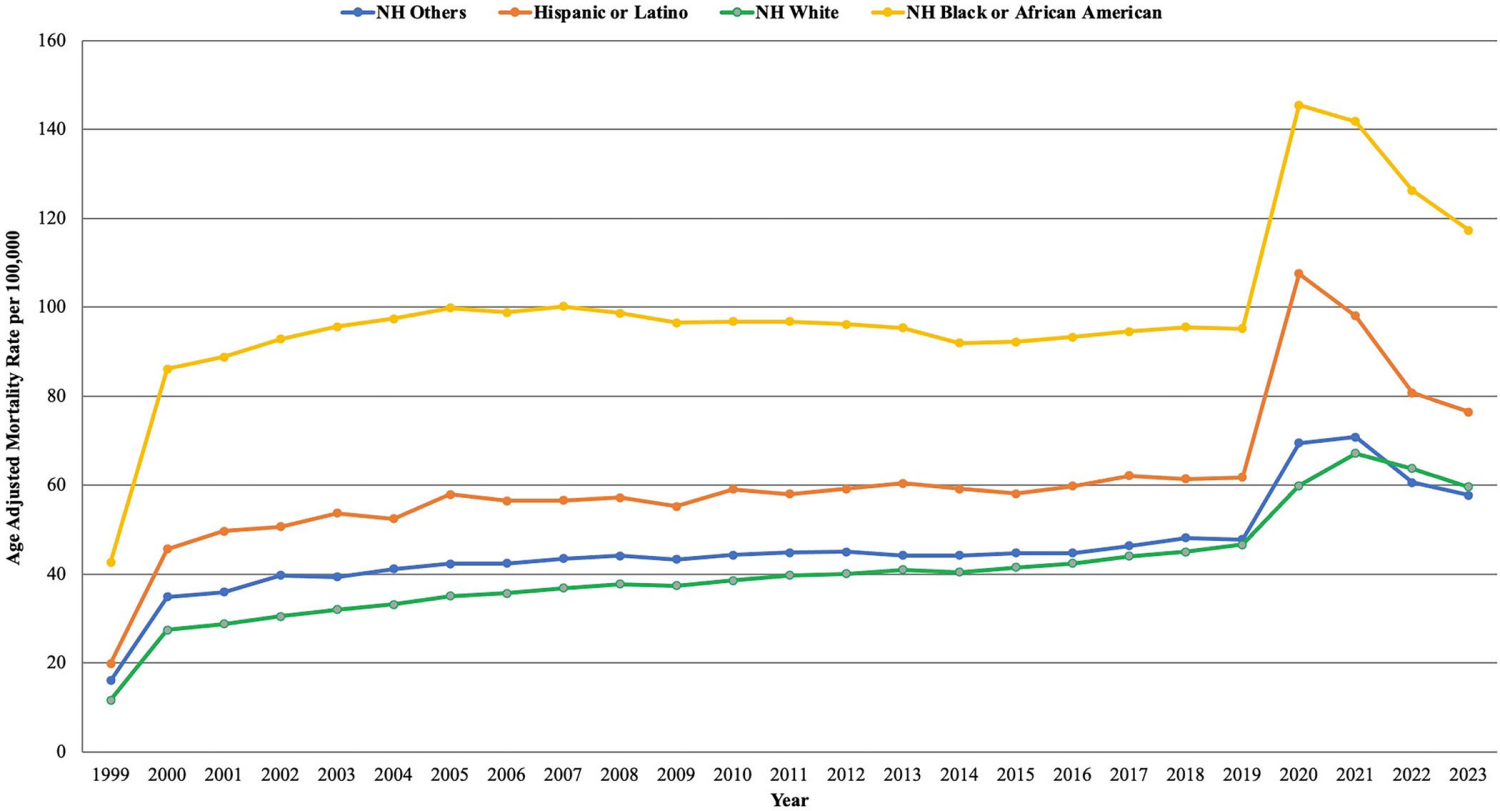
Age‐adjusted mortality rates (AAMRs) per 100 000 individuals stratified by race/ethnicity in the United States, 1999 to 2023.

Similarly, the AAMR for NH White population increased sharply from 11.67 in 1999 to 28.78 in 2001, with an APC of 45.30* (95% CI: 21.31 to 62.20; *p* < 0.000001). The AAMR further increased to 44.99 in 2018, with an APC of 1.98* (95% CI: 1.21 to 2.51; *p* = 0.006). From 2018 to 2021, the AAMR again rose sharply from 44.99 to 67.18, with an APC of 14.66* (95% CI: 9.23 to 18.25; *p* = 0.0076). This was followed by a stable trend till 2023 (Supporting Information S1: Table 2 and Supporting Information S1: Table 4, Figure 2).

### 3.4 DM and HTN‐Related Mortality Trends Stratified by Geographical Location

#### 3.4.1 Census Region

From 1999 to 2023, the Southern region had the highest average AAMR, followed by the West, Midwest, and Northeast.

The Southern region saw a significant rise in AAMR from 14.94 in 1999 to 38.57 in 2001 with an APC of 47.20* (95% CI: 21.67 to 65.58; *p* < 0.000001). This was followed by a further increase to 58.77 in 2018, with an APC of 1.77* (95% CI: 1.03 to 2.30; *p* = 0.0016). The AAMR again rose to 92.66 in 2021, reflecting an APC of 17.77* (95% CI: 12.30 to 21.47; *p* < 0.000001). The AAMR then significantly declined to 80.86 in 2023, with an APC of −7.35* (95% CI: −12.30 to −1.37; *p* = 0.023).

In the West, there was a significant increase from 15.99 in 1999 to 35.51 in 2001, reflecting an APC of 43.01* (95% CI: 20.32 to 58.97; *p* < 0.000001). This was followed by a further increase in AAMR to 50.85 in 2018, with an APC of 1.40* (95% CI: 0.68 to 1.90; *p* = 0.0036). Similarly the AAMR significantly rose to 77.45 in 2021, with an APC of 15.10* (95% CI: 10.01 to 18.36; *p* < 0.000001). From 2021 to 2023 the region saw a significant decline in AAMR from 77.45 to 64.36 in 2023, reflecting an APC of −8.74* (95% CI: −13.59 to −3.09; *p* = 0.0072).

The Midwest region also saw a significant rise in AAMR, increasing from 15.30 in 1999 to 34.61 in 2001 with an APC of 41.74* (95% CI: 17.72 to 58.64; *p* < 0.000001). The AAMR further increased to 49.45 in 2018, reflecting an APC of 1.52* (95% CI: 0.64 to 2.07; *p* = 0.008). This was followed by an increase in AAMR to 72.60 in 2021 with an APC of 14.53* (95% CI: 8.58 to 18.56; *p* = 0.0024). From 2021 to 2023, the region saw a sharp decline in AAMR from 72.6 to 60.72, with an APC of −10.10* (95% CI: −15.71 to −2.89; *p* = 0.015).

**Figure 3:**
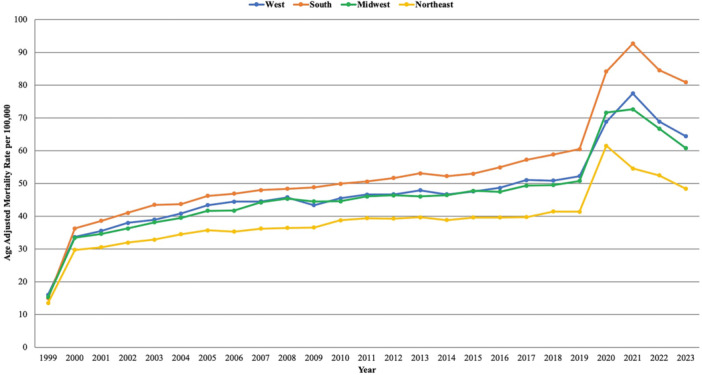
Age‐adjusted mortality rates (AAMRs) per 100 000 individuals stratified by census region in the United States, 1999 to 2023.

Likewise, in the Northeast region there was also a significant rise in AAMR from 13.51 in 1999 to 30.52 in 2001, with an APC of 33.11* (95% CI: 2.79 to 56.94; *p* = 0.0032). This was followed by a stable trend through 2023. (Supporting Information S1: Table 5−6, Figure 3).

#### 3.4.2 Statewide

From 1999 to 2020, the states in the top 90th percentile included Mississippi, District of Columbia, Oklahoma, West Virginia and lastly Texas. Whereas that in the bottom 10th percentile included Montana, Maine, Connecticut, Utah and lastly, Massachusetts. From 2021 to 2023, the states in the top 90th percentile included Oklahoma, Mississippi, South Carolina, West Virginia and lastly, Delaware. The states in the bottom 10th percentile included Utah, New Hampshire, New Jersey, Massachusetts and lastly, Connecticut (Supporting Information S1: Table 5).

#### 3.4.3 Urban‐Rural

From 1999 to 2020, rural areas had a considerably higher average AAMR than urban areas. In Rural areas there was a significant rise in AAMR from 14.26 in 1999 to 35.50 in 2001 with an APC of 46.47* (95% CI: 21.45 to 65.34; *p* < 0.000001). This was followed by a further increase in AAMR to 61.14 in 2018, reflecting an APC of 2.44* (95% CI: 1.52 to 3.00; *p* = 0.0052). The AAMR then increased to 85.59 in 2020 with an APC of 17.95* (95% CI: 8.42 to 25.10; *p* < 0.000001).

**Figure 4:**
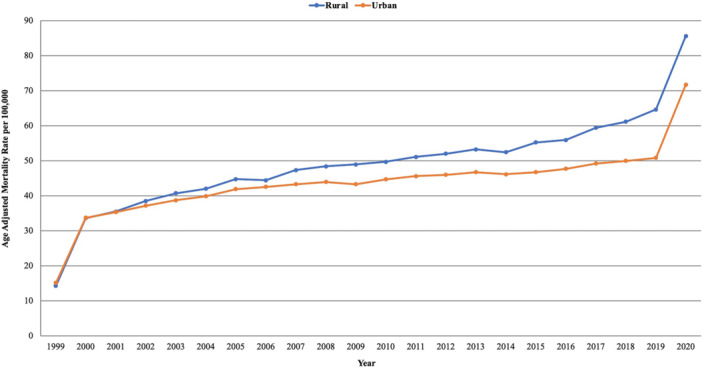
Age‐adjusted mortality rates (AAMRs) per 100 000 individuals stratified by urbanization in the United States, 1999 to 2020. *Data for urbanization AAMRs was unavailable for 2021−2023.

The urban areas also saw a significant increase from 15.08 in 1999 to 35.30 in 2001, reflecting an APC of 43.00* (95% CI: 17.46 to 63.39; *p* < 0.000001). Following this peak, the AAMR further increased to 49.97 in 2018 with an APC of 1.36* (95% CI: 0.33 to 1.95; *p* = 0.019). The AAMR again rose sharply from 49.97 in 2018 to 71.70 in 2020 with an APC of 18.08* (95% CI: 7.45 to 25.43; *p* < 0.000001). (Supporting Information S1: Table 7, Figure 4).

## 4 Discussion

Our analysis highlights a 4.5‐fold increase (AAMR: 14.9 in 1999 vs. 66.8 in 2023) in DM and MI‐related mortality among adults in the United States with substantial temporal variation. The mortality rates peaked during the COVID‐19 pandemic with an AAMR of 77.9 in 2021. Second, males consistently exhibited higher AAMRs compared to females. Third, racial disparities were evident, with NH Black or African American individuals showing the highest AAMRs throughout the study period, followed by Hispanic or Latino individuals, NH Other populations, and NH White individuals. Additionally, regional and geographic disparities were evident, with the Southern region and rural areas consistently reporting higher AAMRs compared to other regions and urban areas. States such as Oklahoma, Mississippi, and West Virginia demonstrated persistently high mortality rates, whereas states like Utah, New Hampshire, and Massachusetts reported much lower rates.

The AAMR for DM and HTN‐related deaths increased sharply from 1999 to 2023. This trend can be attributed to the ongoing obesity epidemic and the increasing prevalence of HTN, hyperlipidemia, smoking, and physical inactivity [8, 18, 28]. Data from the National Health and Nutrition Examination Survey indicate that the management of these modifiable risk factors in patients with DM is suboptimal. Specifically, during the 1988‐2010 survey period, 56.2% of individuals had low‐density lipoprotein cholesterol levels below the recommended target (≤ 100 mg/dL [2.59 mmol/L]), 51.1% had blood pressure readings under the target (≤ 130/80 mmHg), and 52.5% had HbA1c levels within the recommended range (< 7.0% [< 53 mmol/mol]) [23, 29, 30]. In contrast, the steep incline after 2019 aligns with the impact of the COVID‐19 pandemic. The pandemic disproportionately affected individuals with pre‐existing conditions, such as DM and HTN, who were more vulnerable to severe illness and mortality. Additionally, the pandemic exacerbated existing health inequities, strained healthcare systems, and disrupted the management of chronic conditions, contributing further to the surge in deaths [31, 32]. The sharp rise in AAMR during the COVID‐19 pandemic highlights that it disproportionately affected vulnerable populations, further amplifying existing health disparities. Communities already burdened with high rates of DM and HTN such as NH Black Americans, low‐income families, and rural populations were hit particularly hard [32, 33]. These groups often face limited access to healthcare, higher prevalence of underlying conditions, and socioeconomic disadvantages. The pandemic magnified these vulnerabilities, resulting in a marked increase in mortality [34, 35].

Our analysis also shows that males had a marginally higher AAMR than females, consistent with previous studies suggesting that men with DM face a greater mortality risk. This disparity is attributed to factors such as poorer glycemic control, a higher prevalence of cardiovascular complications, and delays in seeking medical care. Additionally, unhealthy behaviors such as smoking and alcohol use, compounded by greater social and competitive pressures, may further elevate mortality risk among men [34, 35].

Significant racial and ethnic disparities in DM and HTN‐related mortality were also evident in our study, with NH Black Americans experiencing the highest AAMR. These inequities stem from a complex interplay of genetic predisposition, socioeconomic disadvantage, healthcare access, structural racism, and cultural differences in diet and lifestyle. For instance, NH Black Americans face higher rates of poverty and limited access to healthcare, which contribute to elevated mortality rates. Moreover, the long‐term financial burden of managing diabetes exacerbates these disparities, placing strain on both healthcare systems and the economic well‐being of affected families [36, 37].

We observed higher mortality rates in the Southern states compared to other regions, as well as in rural versus urban counties. The Southern states exhibited higher AAMR and previous studies sow lower levels of physical activity in these states (Kentucky 16.1%, Mississippi 16.2%, Arkansas 9.4%, Louisiana 19.8%, and Tennessee 22.1%) [38]. Similarly, smoking, HTN, and hypercholesterolemia are more prevalent in these states. Additionally, factors such as social gradients in education, employment, immigration, and demographic behavior patterns are critical in shaping these mortality trends. In this context, the rural‐urban mortality gap has more than doubled over the past two decades, largely due to the disproportionate distribution of socioeconomic and health indicators between rural and urban counties [1, 37, 38, 39].

### 4.1 Limitations

Several limitations should be considered when interpreting our findings. This study utilized data from death certificates, which may have been influenced by co‐existing pathologies, such as cardiovascular diseases, potentially impacting the attribution of deaths to DM and HTN. If healthcare providers completing death certificates fail to include major comorbidities, such as DM or HTN, these contributing factors remain unrecognized in mortality statistics. Additionally, the CDC WONDER database lacks information on trends in life expectancy in the United States, limiting our ability to contextualize the mortality data fully. Moreover, the use of ICD‐10 codes for identifying mortality related to DM and HTN is subject to potential misclassification and variations in coding practices across different periods, which may impact the accuracy of our findings. The mortality records that classify the underlying cause of death based on the primary condition listed on the death certificate can potentially underestimate the contribution of multicausal mortality factors such as DM and HTN, which frequently act as contributors to cardiovascular mortality rather than direct causes. This distinction is crucial for interpreting our results, as patients with DM and HTN may have had other comorbidities influencing mortality outcomes.

## 5 Conclusion

Our study found a 4.5‐fold increase in DM and HTN‐related mortality in the US from 1999 to 2023, with substantial temporal and demographic disparities. Mortality rates peaked during the COVID‐19 pandemic and males consistently exhibited higher mortality rates than females. NH Black individuals experience the highest mortality burden, followed by Hispanic or Latino populations, NH Other groups, and NH White individuals. Geographic disparities were identified, with the Southern region and rural areas demonstrating higher mortality rates compared to urban regions. These findings emphasize the urgent need for targeted public health interventions, equitable healthcare access, and improved management strategies to address the rising burden of DM and HTN‐related mortality in high‐risk populations.

## Author Contributions

Conceptualization, data curation, and project administration were carried out by Mennatalla Ayyad, and Raheel Ahmed. Supervision was carried out by Raheel Ahmed, and Mennatalla Ayyad. Formal analysis of data was carried out by Shahnawaz Hashmi, Iqra Safdar, Eeshal Zulfiqar, Maryam Shahzad, and Sonia Hurjkaliani. Formal analysis, methodology, and software was carried out by Eeshal Zulfiqar, Gabriel Imbianozor, Maryam Shahzad, Sonia Hurjkaliani, Muhammad Hazqeel Kazmi, Iqra Safdar, and Sonia Hurjkaliani. Writing the original draft was carried out by Mennatalla Ayyad, Nimra Zuberi, Toqeer Ahmed, Gauri Balan Sujay, Gabriel Imbianozor, Syeda Hazqah Kazmi, Hamid Talal, and Muhammad Farooq Khan. Writing, reviewing, and editing were carried out by Mennatalla Ayyad and Raheel Ahmed. Visualization and validation were carried out by Eeshal Zulfiqar, Maryam Shahzad, and Sonia Hurjkaliani.

## Ethics Statement

The authors have nothing to report.

## Consent

The authors have nothing to report.

## Conflicts of Interest

The authors declare no conflicts of interest.

## Supporting information

Supplemental_Appendix.

## Data Availability

All data generated or analyzed during this study are included in this article. Further inquiries can be directed to the corresponding author.
