# Supplementary material for: Rising Mortality Related to Diabetes Mellitus and Hypertension: Trends and Disparities in the United States (1999−2023)
Source: Clin Cardiol. 2025 Apr 16;48(4):e70132. doi: 10.1002/clc.70132 (PMC12000922; doi:10.1002/clc.70132)
Supplement: Supplementary file 1 — Supplemental_Appendix. [file CLC-48-e70132-s001.docx]

**Supplementary Appendix**

**Supplemental Table 1: Diabetes Mellitus (DM) and Hypertension (HTN)-Related Deaths, Stratified by Sex and Race in the United States, 1999 to 2023**

| Year | Overall | Female | Male | NH Black or African American | NH White | Hispanic or Latino | NH Others |
| --- | --- | --- | --- | --- | --- | --- | --- |
| 1999 | 26396 | 15315 | 11081 | 6449 | 17371 | 1735 | 749 |
| 2000 | 60214 | 33899 | 26315 | 13098 | 41069 | 4140 | 1747 |
| 2001 | 64252 | 35825 | 28427 | 13791 | 43579 | 4767 | 1922 |
| 2002 | 69116 | 37770 | 31346 | 14724 | 46744 | 5175 | 2241 |
| 2003 | 73507 | 39695 | 33812 | 15494 | 49664 | 5799 | 2349 |
| 2004 | 76830 | 40961 | 35869 | 16085 | 51997 | 5947 | 2580 |
| 2005 | 82564 | 43929 | 38635 | 16943 | 55709 | 6913 | 2823 |
| 2006 | 85087 | 44387 | 40700 | 17223 | 57545 | 7113 | 3003 |
| 2007 | 89108 | 46172 | 42936 | 17913 | 60379 | 7465 | 3216 |
| 2008 | 92586 | 47569 | 45017 | 18115 | 62903 | 7953 | 3394 |
| 2009 | 93394 | 47349 | 46045 | 18224 | 63263 | 8111 | 3541 |
| 2010 | 97757 | 49127 | 48630 | 18751 | 66009 | 8965 | 3760 |
| 2011 | 102687 | 51318 | 51369 | 19436 | 69443 | 9434 | 4140 |
| 2012 | 106235 | 52107 | 54128 | 19941 | 71430 | 10133 | 4417 |
| 2013 | 110602 | 53632 | 56970 | 20459 | 74187 | 10983 | 4644 |
| 2014 | 111798 | 53335 | 58463 | 20467 | 74472 | 11463 | 4972 |
| 2015 | 116666 | 55040 | 61626 | 21222 | 77595 | 11974 | 5352 |
| 2016 | 121531 | 56809 | 64722 | 22158 | 80462 | 12855 | 5630 |
| 2017 | 129080 | 59920 | 69160 | 23214 | 85079 | 14109 | 6210 |
| 2018 | 134212 | 61224 | 72988 | 24064 | 88461 | 14519 | 6738 |
| 2019 | 140358 | 63571 | 76787 | 24647 | 93125 | 15220 | 7013 |
| 2020 | 199038 | 89047 | 109991 | 38798 | 121048 | 27875 | 10732 |
| 2021 | 206069 | 91402 | 114667 | 37236 | 130564 | 26121 | 10621 |
| 2022 | 196198 | 87701 | 108497 | 33805 | 129009 | 22091 | 9702 |
| 2023 | 183833 | 81580 | 102253 | 31303 | 120866 | 20847 | 9182 |

**Supplemental Table 2: Annual Percent Change (APC) of Diabetes Mellitus (DM) and Hypertension (HTN)-Related Age-Adjusted Mortality Rates per 100,000 in the United States, 1999 to 2023**

| Year Interval | APC (95% CI) |
| --- | --- |
| Overall |  |
| 1999–2001 | 43.98 (19.87 to 61.06) |
| 2001–2018 | 1.60 (0.85 to 2.1) |
| 2018–2021 | 15.71 (10.33 to 19.33) |
| 2021–2023 | -8.44 (-13.54 to -2.19) |
| Male |  |
| 1999–2001 | 45.80* (19.05 to 65.16) |
| 2001–2018 | 2.38* (1.58 to 2.94) |
| 2018–2021 | 16.06* (10.82 to 19.64) |
| 2021–2023 | 7.93* (-12.80 to -1.89) |
| Female |  |
| 1999–2001 | 40.18* (17.30 to 56.18) |
| 2001–2018 | 0.74 (-0.04 to 1.25) |
| 2018–2021 | 15.19* (9.35 to 19.04) |
| 2021–2023 | -8.95* (-14.42 to -2.00) |
| NH Black or African American |  |
| 1999–2001 | 37.95* (13.22 to 55.25) |
| 2001–2018 | -0.30 (-1.27 to 0.29) |
| 2018–2021 | 16.66* (9.85 to 21.35) |
| 2021–2023 | -11.97* (-18.06 to -4.30) |
| NH White |  |
| 1999–2001 | 45.30* (21.31 to 62.20) |
| 2001–2018 | 1.98* (1.20 to 2.51) |
| 2018–2021 | 14.66* (9.23 to 18.25) |
| 2021–2023 | -5.54 (-10.79 to 1.07) |
| Hispanic or Latino |  |
| 1999–2001 | 43.97* (2.83 to 79.11) |
| 2001–2018 | 0.88 (-3.89 to 1.76) |
| 2018–2021 | 19.56* (11.17 to 26.19) |
| 2021–2023 | -16.80* (-24.06 to -6.80) |
| NH Others |  |
| 1999–2001 | 41.26* (10.68 to 65.24) |
| 2001–2018 | 0.84 (-0.21 to 1.46) |
| 2018–2021 | 15.88* (10.31 to 19.65) |
| 2021–2023 | -11.92* (-17.07 to -5.85) |
| Northeast |  |
| 1999–2001 | 33.11* (2.79 to 56.94) |
| 2001–2023 | 2.30 (-0.06 to 3.11) |
| Midwest |  |
| 1999–2001 | 41.74* (17.72 to 58.64) |
| 2001–2018 | 1.52* (0.64 to 2.07) |
| 2018–2021 | 14.53* (8.58 to 18.56) |
| 2021–2023 | -10.10* (-15.71 to -2.89) |
| South |  |
| 1999–2001 | 47.20* (21.67 to 65.58) |
| 2001–2018 | 1.77* (1.03 to 2.30) |
| 2018–2021 | 17.77* (12.30 to 21.47) |
| 2021–2023 | -7.35* (-12.30 to -1.37) |
| West |  |
| 1999–2001 | 43.01* (20.32 to 58.97) |
| 2001–2018 | 1.40* (0.68 to 1.90) |
| 2018–2021 | 15.10* (10.01 to 18.36) |
| 2021–2023 | -8.74* (-13.59 to -3.09) |
| Urban |  |
| 1999–2001 | 43.00* (17.46 to 63.39) |
| 2001–2018 | 1.36* (0.33 to 1.95) |
| 2018-2020 | 18.08* (7.45 to 25.43) |
| Rural |  |
| 1999–2001 | 46.47* (21.45 to 65.34) |
| 2001–2018 | 2.44* (1.52 to 3.00) |
| 2018-2020 | 17.95* (8.42 to 25.09) |
| Ten-year Age groups* |  |
| 25–34 years |  |
| 1999–2003 | 24.62* (13.74 to 46.05) |
| 2003–2018 | 2.38* (0.61 to 3.42) |
| 2018–2021 | 20.17* (11.87 to 25.27) |
| 2021–2023 | -13.79* (-21.75 to -3.45) |
| 35–44 years |  |
| 1999–2001 | 41.54* (17.15 to 63.73) |
| 2001–2018 | 3.59* (2.44 to 4.23) |
| 2018–2021 | 18.35* (11.21 to 22.55) |
| 2021–2023 | -14.81* (-21.66 to -7.01) |
| 45–54 years |  |
| 1999–2001 | 41.81* (18.11 to 61.84) |
| 2001–2018 | 3.04* (2.13 to 3.59) |
| 2018–2021 | 16.92* (10.85 to 20.69) |
| 2021–2023 | -13.21* (-19.37 to -6.49) |
| 55–64 years |  |
| 1999–2001 | 38.10* (14.82 to 56.39) |
| 2001–2018 | 1.77* (1.02 to 2.29) |
| 2018–2021 | 17.18* (11.78 to 20.75) |
| 2021–2023 | -11.80* (-16.74 to -5.83) |
| 65–74 years |  |
| 1999–2001 | 39.46* (16.06 to 55.67) |
| 2001–2018 | 0.84* (0.06 to 1.38) |
| 2018–2021 | 16.97* (11.38 to 20.85) |
| 2021–2023 | -10.22* (-15.20 to -3.92) |
| 75–84 years |  |
| 1999–2001 | 45.08* (20.13 to 62.94) |
| 2001–2018 | 1.24* (0.38 to 1.81) |
| 2018–2021 | 14.76* (8.50 to 18.77) |
| 2021–2023 | -6.45 (-12.06 to 1.03) |
| 85+ years |  |
| 1999–2001 | 48.40* (21.33 to 70.13) |
| 2001–2018 | 2.24* (1.38 to 2.82) |
| 2018–2021 | 14.18* (8.57 to 17.62) |
| 2021–2023 | -5.53 (-11.25 to 1.63) |

*****For ten-year age groups, crude mortality rates were used.

**Supplemental Table 3: Overall and Sex‐Stratified DM and HTN-Related Age-Adjusted Mortality Rates per 100,000 in the United States, 1999 to 2023**

| Age-Adjusted Rate (95% CI) | | | |
| --- | --- | --- | --- |
| Year | **Male** | **Female** | **Overall** |
| 1999 | 15.27 (14.98 - 15.56) | 14.54 (14.31 - 14.77) | 14.94 (14.76 - 15.12) |
| 2000 | 35.78 (35.34 - 36.22) | 31.78 (31.44 - 32.12) | 33.67 (33.4 - 33.94) |
| 2001 | 37.82 (37.37 - 38.26) | 33.05 (32.71 - 33.4) | 35.35 (35.07 - 35.62) |
| 2002 | 40.94 (40.48 - 41.4) | 34.45 (34.1 - 34.79) | 37.4 (37.13 - 37.68) |
| 2003 | 43.12 (42.65 - 43.59) | 35.74 (35.39 - 36.1) | 39.11 (38.83 - 39.39) |
| 2004 | 44.92 (44.45 - 45.39) | 36.41 (36.05 - 36.76) | 40.24 (39.96 - 40.53) |
| 2005 | 47.25 (46.77 - 47.73) | 38.38 (38.02 - 38.75) | 42.41 (42.12 - 42.7) |
| 2006 | 48.6 (48.12 - 49.08) | 38.15 (37.79 - 38.51) | 42.83 (42.54 - 43.12) |
| 2007 | 50.13 (49.64 - 50.61) | 38.97 (38.61 - 39.32) | 44.02 (43.73 - 44.31) |
| 2008 | 51.3 (50.82 - 51.78) | 39.37 (39.01 - 39.73) | 44.74 (44.45 - 45.03) |
| 2009 | 51.14 (50.67 - 51.62) | 38.58 (38.23 - 38.93) | 44.25 (43.96 - 44.53) |
| 2010 | 53.13 (52.65 - 53.62) | 39.41 (39.06 - 39.76) | 45.52 (45.24 - 45.81) |
| 2011 | 54.3 (53.82 - 54.78) | 40.19 (39.84 - 40.55) | 46.51 (46.23 - 46.8) |
| 2012 | 55.62 (55.14 - 56.1) | 39.88 (39.53 - 40.23) | 46.99 (46.71 - 47.28) |
| 2013 | 56.91 (56.43 - 57.38) | 40.33 (39.98 - 40.68) | 47.81 (47.52 - 48.09) |
| 2014 | 56.63 (56.16 - 57.1) | 39.34 (39 - 39.68) | 47.14 (46.86 - 47.42) |
| 2015 | 58.27 (57.8 - 58.74) | 39.78 (39.44 - 40.11) | 48.12 (47.84 - 48.4) |
| 2016 | 59.74 (59.27 - 60.21) | 40.29 (39.95 - 40.63) | 49.04 (48.76 - 49.32) |
| 2017 | 62.22 (61.74 - 62.69) | 41.53 (41.19 - 41.87) | 50.85 (50.57 - 51.13) |
| 2018 | 64.13 (63.66 - 64.61) | 41.66 (41.33 - 42) | 51.79 (51.51 - 52.07) |
| 2019 | 65.9 (65.43 - 66.38) | 42.41 (42.08 - 42.75) | 53.02 (52.74 - 53.3) |
| 2020 | 92.34 (91.78 - 92.89) | 58.64 (58.25 - 59.04) | 73.88 (73.55 - 74.2) |
| 2021 | 96.86 (96.29 - 97.44) | 62.23 (61.82 - 62.64) | 77.93 (77.59 - 78.27) |
| 2022 | 89.35 (88.8 - 89.89) | 56.64 (56.26 - 57.02) | 71.3 (70.98 - 71.62) |
| 2023 | 84.58 (84.05 - 85.11) | 52.61 (52.25 - 52.98) | 66.88 (66.57 - 67.19) |

**Supplemental Table 4: Diabetes Mellitus (DM) and Hypertension (HTN)-Related Age-Adjusted Mortality Rates per 100,000, Stratified by Race in the United States, 1999 to 2023**

|  | Age-Adjusted Rate (95% CI) | | | |
| --- | --- | --- | --- | --- |
| Year | **NH Black or African American** | **NH White** | **Hispanic or Latino** | **NH Others** |
| 1999 | 42.64 (41.59 - 43.69) | 11.67 (11.5 - 11.84) | 19.91 (18.93 - 20.89) | 16 (14.8 - 17.21) |
| 2000 | 86.09 (84.59 - 87.58) | 27.43 (27.17 - 27.7) | 45.65 (44.2 - 47.1) | 34.88 (33.17 - 36.58) |
| 2001 | 88.76 (87.26 - 90.26) | 28.78 (28.51 - 29.05) | 49.62 (48.15 - 51.09) | 35.91 (34.24 - 37.58) |
| 2002 | 92.82 (91.29 - 94.34) | 30.51 (30.23 - 30.78) | 50.66 (49.22 - 52.1) | 39.72 (38.01 - 41.43) |
| 2003 | 95.62 (94.09 - 97.15) | 32.02 (31.74 - 32.3) | 53.7 (52.26 - 55.15) | 39.38 (37.73 - 41.04) |
| 2004 | 97.36 (95.82 - 98.89) | 33.12 (32.83 - 33.4) | 52.41 (51.02 - 53.8) | 41.11 (39.46 - 42.76) |
| 2005 | 99.83 (98.29 - 101.36) | 35.03 (34.74 - 35.32) | 57.92 (56.5 - 59.34) | 42.35 (40.73 - 43.97) |
| 2006 | 98.82 (97.31 - 100.33) | 35.63 (35.34 - 35.92) | 56.5 (55.13 - 57.87) | 42.4 (40.82 - 43.97) |
| 2007 | 100.18 (98.68 - 101.68) | 36.83 (36.54 - 37.13) | 56.55 (55.21 - 57.88) | 43.47 (41.92 - 45.03) |
| 2008 | 98.65 (97.18 - 100.13) | 37.71 (37.41 - 38.01) | 57.22 (55.91 - 58.53) | 44.08 (42.55 - 45.61) |
| 2009 | 96.49 (95.05 - 97.93) | 37.4 (37.1 - 37.69) | 55.23 (53.98 - 56.48) | 43.26 (41.79 - 44.73) |
| 2010 | 96.7 (95.28 - 98.13) | 38.5 (38.21 - 38.8) | 59.08 (57.8 - 60.35) | 44.25 (42.79 - 45.7) |
| 2011 | 96.77 (95.37 - 98.17) | 39.73 (39.43 - 40.03) | 58.01 (56.8 - 59.23) | 44.78 (43.37 - 46.18) |
| 2012 | 96.11 (94.74 - 97.49) | 40.1 (39.8 - 40.39) | 59.17 (57.98 - 60.36) | 44.98 (43.62 - 46.34) |
| 2013 | 95.28 (93.93 - 96.62) | 40.95 (40.65 - 41.25) | 60.39 (59.22 - 61.56) | 44.23 (42.93 - 45.53) |
| 2014 | 91.9 (90.6 - 93.2) | 40.43 (40.14 - 40.73) | 59.15 (58.02 - 60.27) | 44.23 (42.98 - 45.49) |
| 2015 | 92.21 (90.93 - 93.48) | 41.51 (41.22 - 41.81) | 58.07 (56.99 - 59.14) | 44.74 (43.52 - 45.97) |
| 2016 | 93.23 (91.97 - 94.5) | 42.36 (42.07 - 42.66) | 59.81 (58.74 - 60.88) | 44.74 (43.55 - 45.93) |
| 2017 | 94.5 (93.25 - 95.75) | 44.03 (43.72 - 44.33) | 62.12 (61.06 - 63.18) | 46.36 (45.18 - 47.53) |
| 2018 | 95.45 (94.21 - 96.69) | 44.99 (44.69 - 45.3) | 61.44 (60.41 - 62.47) | 48.1 (46.94 - 49.27) |
| 2019 | 95.11 (93.89 - 96.33) | 46.65 (46.34 - 46.95) | 61.77 (60.76 - 62.78) | 47.8 (46.66 - 48.93) |
| 2020 | 145.44 (143.95 - 146.93) | 59.91 (59.56 - 60.25) | 107.48 (106.18 - 108.78) | 69.5 (68.16 - 70.83) |
| 2021 | 141.73 (140.24 - 143.22) | 67.18 (66.8 - 67.55) | 97.96 (96.72 - 99.19) | 70.86 (69.49 - 72.22) |
| 2022 | 126.26 (124.87 - 127.64) | 63.71 (63.36 - 64.07) | 80.72 (79.62 - 81.82) | 60.57 (59.35 - 61.79) |
| 2023 | 117.24 (115.91 - 118.58) | 59.61 (59.27 - 59.95) | 76.38 (75.31 - 77.45) | 57.74 (56.55 - 58.93) |

**Supplemental Table 5: Diabetes Mellitus (DM) and Hypertension (HTN)-Related Age-Adjusted Mortality Rates per 100,000, Stratified by States in the United States, 1999 to 2023**

| State | Age-Adjusted Rate (95% CI) | |
| --- | --- | --- |
|  | **1999-2020** | **2021-2023** |
| Alabama | 41.96 (41.5 - 42.43) | 57.7 (56.3 - 59) |
| Alaska | 33.33 (31.85 - 34.82) | 45.5 (41.8 - 49.2) |
| Arizona | 32.66 (32.31 - 33.02) | 64.4 (63.2 - 65.6) |
| Arkansas | 47.06 (46.44 - 47.68) | 92.1 (89.9 - 94.3) |
| California | 53.78 (53.58 - 53.98) | 73.3 (72.7 - 73.8) |
| Colorado | 34.95 (34.49 - 35.4) | 69.6 (68.1 - 71.1) |
| Connecticut | 27.02 (26.61 - 27.43) | 30.7 (29.5 - 31.8) |
| Delaware | 43.73 (42.65 - 44.8) | 104 (100.1 - 107.9) |
| District of Columbia | 86.19 (84.21 - 88.18) | 103.3 (97.8 - 108.9) |
| Florida | 33.4 (33.21 - 33.59) | 54.7 (54.1 - 55.2) |
| Georgia | 46.34 (45.95 - 46.72) | 82.6 (81.4 - 83.8) |
| Hawaii | 46.31 (45.43 - 47.2) | 58.3 (55.9 - 60.7) |
| Idaho | 36.88 (36.08 - 37.67) | 73.7 (71.1 - 76.3) |
| Illinois | 36.79 (36.52 - 37.06) | 50.1 (49.3 - 50.9) |
| Indiana | 45.84 (45.42 - 46.26) | 72.2 (70.9 - 73.5) |
| Iowa | 41.14 (40.6 - 41.68) | 76.1 (74.2 - 78.1) |
| Kansas | 32.11 (31.59 - 32.63) | 58.2 (56.4 - 60) |
| Kentucky | 49.43 (48.89 - 49.96) | 95.9 (94.1 - 97.8) |
| Louisiana | 54.78 (54.21 - 55.34) | 98.4 (96.5 - 100.3) |
| Maine | 29.58 (28.9 - 30.27) | 49.7 (47.5 - 51.9) |
| Maryland | 54.49 (53.99 - 54.98) | 86.5 (85 - 88) |
| Massachusetts | 24.12 (23.83 - 24.41) | 34.7 (33.9 - 35.6) |
| Michigan | 43.85 (43.53 - 44.18) | 61 (60.1 - 62) |
| Minnesota | 44.71 (44.25 - 45.16) | 86.3 (84.8 - 87.9) |
| Mississippi | 86.48 (85.61 - 87.36) | 166.9 (163.8 - 169.9) |
| Missouri | 39.39 (39 - 39.79) | 57.6 (56.4 - 58.8) |
| Montana | 31.28 (30.43 - 32.12) | 60.6 (57.7 - 63.5) |
| Nebraska | 44.39 (43.63 - 45.15) | 83.9 (81.3 - 86.6) |
| Nevada | 33.88 (33.28 - 34.48) | 76.8 (74.8 - 78.9) |
| New Hampshire | 33.36 (32.58 - 34.13) | 45.1 (43 - 47.3) |
| New Jersey | 36.04 (35.72 - 36.35) | 42.3 (41.4 - 43.1) |
| New Mexico | 40.16 (39.45 - 40.87) | 73.5 (71.2 - 75.8) |
| New York | 42.85 (42.62 - 43.08) | 61.3 (60.6 - 62) |
| North Carolina | 53.43 (53.05 - 53.81) | 70.1 (69.1 - 71.2) |
| North Dakota | 44.4 (43.2 - 45.6) | 58.2 (54.7 - 61.8) |
| Ohio | 60.22 (59.87 - 60.57) | 72.5 (71.5 - 73.5) |
| Oklahoma | 81.67 (80.93 - 82.41) | 191.9 (189 - 194.8) |
| Oregon | 44.79 (44.26 - 45.31) | 79.4 (77.7 - 81.1) |
| Pennsylvania | 39.37 (39.11 - 39.62) | 55.3 (54.6 - 56.1) |
| Rhode Island | 45.9 (44.92 - 46.87) | 85.5 (82.1 - 88.8) |
| South Carolina | 57.78 (57.22 - 58.34) | 132.9 (130.9 - 134.9) |
| South Dakota | 42.75 (41.67 - 43.83) | 79.6 (75.7 - 83.4) |
| Tennessee | 56.61 (56.13 - 57.08) | 96.8 (95.3 - 98.3) |
| Texas | 61.61 (61.34 - 61.89) | 97.5 (96.7 - 98.3) |
| Utah | 26.78 (26.19 - 27.38) | 45.2 (43.4 - 47) |
| Vermont | 58.56 (57.11 - 60.02) | 77 (73 - 81.1) |
| Virginia | 38.18 (37.82 - 38.54) | 61.5 (60.4 - 62.5) |
| Washington | 41.72 (41.32 - 42.13) | 65.8 (64.5 - 67) |
| West Virginia | 63.53 (62.68 - 64.39) | 104.4 (101.5 - 107.3) |
| Wisconsin | 38.95 (38.55 - 39.35) | 75.4 (74 - 76.8) |
| Wyoming | 36.25 (34.93 - 37.56) | 79.3 (74.5 - 84) |
|  |  |  |

**Supplemental Table 6: Diabetes Mellitus (DM) and Hypertension (HTN)-Related Age-Adjusted Mortality Rates per 100,000, Stratified by Census Region in the United States, 1999 to 2023**

| Census Region | Year | Age-Adjusted Rate (95% CI) |
| --- | --- | --- |
| Northeast |  |  |
| Northeast | 1999 | 13.51 (13.14 - 13.89) |
| Northeast | 2000 | 29.67 (29.12 - 30.23) |
| Northeast | 2001 | 30.52 (29.97 - 31.08) |
| Northeast | 2002 | 31.94 (31.38 - 32.51) |
| Northeast | 2003 | 32.84 (32.27 - 33.41) |
| Northeast | 2004 | 34.48 (33.9 - 35.06) |
| Northeast | 2005 | 35.67 (35.08 - 36.26) |
| Northeast | 2006 | 35.26 (34.67 - 35.84) |
| Northeast | 2007 | 36.15 (35.56 - 36.74) |
| Northeast | 2008 | 36.41 (35.82 - 36.99) |
| Northeast | 2009 | 36.51 (35.93 - 37.1) |
| Northeast | 2010 | 38.76 (38.16 - 39.36) |
| Northeast | 2011 | 39.37 (38.77 - 39.97) |
| Northeast | 2012 | 39.24 (38.65 - 39.83) |
| Northeast | 2013 | 39.65 (39.06 - 40.24) |
| Northeast | 2014 | 38.79 (38.2 - 39.37) |
| Northeast | 2015 | 39.6 (39.01 - 40.18) |
| Northeast | 2016 | 39.59 (39.01 - 40.18) |
| Northeast | 2017 | 39.71 (39.13 - 40.28) |
| Northeast | 2018 | 41.38 (40.79 - 41.96) |
| Northeast | 2019 | 41.34 (40.76 - 41.92) |
| Northeast | 2020 | 61.48 (60.77 - 62.18) |
| Northeast | 2021 | 54.5 (53.84 - 55.16) |
| Northeast | 2022 | 52.41 (51.78 - 53.05) |
| Northeast | 2023 | 48.33 (47.72 - 48.94) |
| Midwest |  |  |
| Midwest | 1999 | 15.3 (14.93 - 15.68) |
| Midwest | 2000 | 33.4 (32.85 - 33.95) |
| Midwest | 2001 | 34.61 (34.06 - 35.17) |
| Midwest | 2002 | 36.24 (35.67 - 36.8) |
| Midwest | 2003 | 38.09 (37.52 - 38.67) |
| Midwest | 2004 | 39.48 (38.9 - 40.07) |
| Midwest | 2005 | 41.62 (41.02 - 42.21) |
| Midwest | 2006 | 41.7 (41.11 - 42.29) |
| Midwest | 2007 | 44.16 (43.56 - 44.77) |
| Midwest | 2008 | 45.31 (44.7 - 45.92) |
| Midwest | 2009 | 44.48 (43.88 - 45.08) |
| Midwest | 2010 | 44.5 (43.91 - 45.1) |
| Midwest | 2011 | 45.98 (45.38 - 46.58) |
| Midwest | 2012 | 46.37 (45.77 - 46.97) |
| Midwest | 2013 | 46.03 (45.44 - 46.62) |
| Midwest | 2014 | 46.38 (45.79 - 46.97) |
| Midwest | 2015 | 47.7 (47.11 - 48.3) |
| Midwest | 2016 | 47.45 (46.86 - 48.05) |
| Midwest | 2017 | 49.29 (48.69 - 49.89) |
| Midwest | 2018 | 49.45 (48.86 - 50.04) |
| Midwest | 2019 | 50.73 (50.14 - 51.33) |
| Midwest | 2020 | 71.56 (70.86 - 72.26) |
| Midwest | 2021 | 72.6 (71.88 - 73.32) |
| Midwest | 2022 | 66.67 (66 - 67.35) |
| Midwest | 2023 | 60.72 (60.07 - 61.36) |
| South |  |  |
| South | 1999 | 14.94 (14.64 - 15.24) |
| South | 2000 | 36.26 (35.79 - 36.73) |
| South | 2001 | 38.57 (38.09 - 39.05) |
| South | 2002 | 40.99 (40.5 - 41.48) |
| South | 2003 | 43.44 (42.94 - 43.94) |
| South | 2004 | 43.68 (43.18 - 44.18) |
| South | 2005 | 46.16 (45.66 - 46.67) |
| South | 2006 | 46.83 (46.33 - 47.34) |
| South | 2007 | 47.92 (47.42 - 48.43) |
| South | 2008 | 48.31 (47.8 - 48.81) |
| South | 2009 | 48.8 (48.3 - 49.29) |
| South | 2010 | 49.89 (49.39 - 50.39) |
| South | 2011 | 50.56 (50.06 - 51.05) |
| South | 2012 | 51.61 (51.11 - 52.1) |
| South | 2013 | 53.05 (52.55 - 53.54) |
| South | 2014 | 52.18 (51.69 - 52.66) |
| South | 2015 | 52.93 (52.45 - 53.41) |
| South | 2016 | 54.89 (54.4 - 55.37) |
| South | 2017 | 57.2 (56.72 - 57.69) |
| South | 2018 | 58.77 (58.29 - 59.26) |
| South | 2019 | 60.46 (59.97 - 60.95) |
| South | 2020 | 84.13 (83.56 - 84.7) |
| South | 2021 | 92.66 (92.06 - 93.27) |
| South | 2022 | 84.51 (83.94 - 85.08) |
| South | 2023 | 80.86 (80.3 - 81.41) |
| West |  |  |
| West | 1999 | 15.99 (15.57 - 16.41) |
| West | 2000 | 33.61 (33.01 - 34.21) |
| West | 2001 | 35.51 (34.9 - 36.12) |
| West | 2002 | 37.96 (37.34 - 38.59) |
| West | 2003 | 38.92 (38.3 - 39.55) |
| West | 2004 | 40.77 (40.13 - 41.4) |
| West | 2005 | 43.33 (42.68 - 43.97) |
| West | 2006 | 44.42 (43.77 - 45.06) |
| West | 2007 | 44.47 (43.83 - 45.11) |
| West | 2008 | 45.73 (45.09 - 46.37) |
| West | 2009 | 43.33 (42.72 - 43.95) |
| West | 2010 | 45.45 (44.82 - 46.07) |
| West | 2011 | 46.6 (45.98 - 47.22) |
| West | 2012 | 46.64 (46.02 - 47.25) |
| West | 2013 | 47.9 (47.29 - 48.52) |
| West | 2014 | 46.64 (46.05 - 47.24) |
| West | 2015 | 47.52 (46.93 - 48.11) |
| West | 2016 | 48.61 (48.02 - 49.2) |
| West | 2017 | 50.98 (50.38 - 51.58) |
| West | 2018 | 50.85 (50.26 - 51.44) |
| West | 2019 | 52.17 (51.58 - 52.76) |
| West | 2020 | 68.81 (68.14 - 69.47) |
| West | 2021 | 77.45 (76.73 - 78.17) |
| West | 2022 | 68.85 (68.19 - 69.51) |
| West | 2023 | 64.36 (63.72 - 65) |

**Supplemental Table 7: Diabetes Mellitus (DM) and Hypertension (HTN)-Related Age-Adjusted Mortality Rates per 100,000 in the United States, Stratified by Urban-Rural Classification, 1999 to 2020**

|  | Age-Adjusted Rate (95% CI) | |
| --- | --- | --- |
| Year | **Urban** | **Rural** |
| 1999 | 15.08 (14.88 - 15.28) | 14.26 (13.85 - 14.66) |
| 2000 | 33.68 (33.38 - 33.97) | 33.57 (32.95 - 34.19) |
| 2001 | 35.3 (35 - 35.61) | 35.5 (34.87 - 36.13) |
| 2002 | 37.14 (36.83 - 37.45) | 38.51 (37.85 - 39.17) |
| 2003 | 38.73 (38.42 - 39.05) | 40.68 (40.01 - 41.35) |
| 2004 | 39.83 (39.52 - 40.15) | 42.01 (41.33 - 42.69) |
| 2005 | 41.9 (41.59 - 42.22) | 44.73 (44.03 - 45.42) |
| 2006 | 42.52 (42.2 - 42.84) | 44.42 (43.73 - 45.11) |
| 2007 | 43.26 (42.94 - 43.58) | 47.33 (46.62 - 48.04) |
| 2008 | 43.94 (43.62 - 44.26) | 48.41 (47.7 - 49.13) |
| 2009 | 43.27 (42.96 - 43.58) | 48.91 (48.2 - 49.63) |
| 2010 | 44.68 (44.37 - 45) | 49.66 (48.94 - 50.37) |
| 2011 | 45.61 (45.3 - 45.93) | 51.1 (50.38 - 51.83) |
| 2012 | 45.98 (45.66 - 46.29) | 51.98 (51.25 - 52.7) |
| 2013 | 46.71 (46.4 - 47.02) | 53.23 (52.5 - 53.96) |
| 2014 | 46.12 (45.81 - 46.42) | 52.43 (51.71 - 53.15) |
| 2015 | 46.7 (46.4 - 47.01) | 55.21 (54.48 - 55.95) |
| 2016 | 47.71 (47.41 - 48.02) | 55.89 (55.15 - 56.62) |
| 2017 | 49.19 (48.88 - 49.49) | 59.39 (58.64 - 60.15) |
| 2018 | 49.97 (49.67 - 50.28) | 61.14 (60.38 - 61.9) |
| 2019 | 50.82 (50.52 - 51.13) | 64.6 (63.83 - 65.38) |
| 2020 | 71.7 (71.35 - 72.05) | 85.59 (84.7 - 86.48) |
